# Online conferences for better learning

**DOI:** 10.1002/ece3.6923

**Published:** 2020-10-22

**Authors:** Christopher J. Lortie

**Affiliations:** ^1^ Department of Biology York University Toronto ON Canada

**Keywords:** conferences, distributed, diversity, equity, inclusivity, learning, networking, online, open science, process, simple rules, technology

## Abstract

Conferences provide an invaluable set of opportunities for professional development. Online, virtual, and distributed conferences do not necessarily mean less opportunity for growth and innovation in science but varied and novel options for communicating the scientific process. Open science and many existing tools are in place in the practice of contemporary ecology and evolution to provide latitude for a much broader scope of sharing and thus learning from conferences. A brief overview of the science supporting online conferences and a highlight of some of the open science concepts in ecology and evolution are provided here to enable better learning through better planning for online conferences.

## Graphical abstract

Online conferences are a necessity. We can use this an opportunity in ecology and evolution to enable better learning and affect social good.

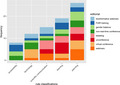

## 1 INTRODUCTION

A crisis is never welcomed, and we must now rally to innovate how we do science. We need to work harder to make conferences better and not only address the immediate challenges but tackle pre‐existing limitations associated with conferences and meetings. Conferences are a profoundly useful mechanism to enable scientific progress as a community. These conventions provide the opportunity to network, collaborate, communicate, connect early‐career researchers with new (and established) collaborators, and most importantly co‐learn and build community. Ecologists and evolutionary biologists are scientists, citizens, educators, and a collective that can affect social good. Hence, conferences allow us to convene and share and learn in different and extended ways (Cooke et al., 2017). These meetings are typically about more than scientific content and facilitate the identification of research gaps and opportunities (Kochetkov et al., 2020; Oester et al., 2017). In some respects, coming together also helps us decide how we feel about the global grand challenges that we face environmentally and as a species (Acocella, 2015). We build collective strength and cohesion. Conferences in ecology and evolution are also incredibly frequent. A brief search on sites that compile conference listings for ecology and evolution including the Nature Ecology and Evolution website, relevant society sites, and The World Academy of Science, Engineering, and Technology (filtered using the key term “ecology”) suggest that there were approximately 400 meetings scheduled for the calendar year of 2020 (Lortie, 2020c). However, as citizens, we have a moral responsibility to flatten the curve of a pandemic through reduced in‐person interactions, smaller conventions only for critical needs, and much less travel to mitigate spread and reduce risks for everyone (Khoo & Lantos, 2020; Verma et al., 2020). More broadly, we can reconsider the carbon costs and other impacts including accessibility and the equity of costs to convene exclusively through face‐to‐face meetings (Julsrud et al., 2014; Neugebauer et al., 2020). In doing so, we have an opportunity to promote equity, diversity, and inclusivity through different planning, by reducing some of the financial costs, and by sharing our insights to wider, more varied communities (Ediger et al., 2010; Montgomery et al., 2012). Even more importantly, we have an incentive to experiment. After all, we are scientists, and all large meetings come with benefits but also challenges. Navigating a large convention (although an app helps), rushing between talks, choosing between concurrent sessions, relatively high carbon footprint for many attendees (Neugebauer et al., 2020), other costs of attendance including fatigue (Julsrud et al., 2014), and now a further moral imperative suggest that we must consider alternatives. The meeting theme of the British Ecological Society, “Ecology Across Borders” and many other contemporary conferences are also ideally suited to design thinking and experimentation (Burdick & Willis, 2011). I propose that we test as many new ways to meet online this year (and next) as possible so as to better align our values and practices including social equity (Parrott, 2017) and open science (Allen & Mehler, 2019; Lowndes et al., 2017) with how we meet. Better learning in particular can be enabled by planning specifically for online and distributed conferences.

## 2 SUPPORTING RESEARCH ON CONFERENCES

The goal here was to briefly explore the learning and networking research associated with online conferences and webinars primarily for organizers and planners within our community. Meetings in ecology and evolution include many activities directly associated with learning and professional development. Breakout meetings at a conference by ecological network partnerships, workshops for skills development, special organized sessions, and discussions by subject matter‐oriented society sections promote skill and expertise development. Networking can be enabled through social mixers, exhibits by NPOs and journals, and explicit events and booths that build professional networks and explore employment opportunities. To ensure that these resources are available for planners and presenters via online meetings and to ensure that the innovations for conferences proposed herein are novel and well‐situated in a framework of evidence‐informed principles from research (Tennant et al., 2016; Tranfield et al., 2003), an overview of key findings is described. There are at least 35 relevant studies that examined the meta‐science of online conferences and networking benefits estimated from a query and analysis of The Web of Science bibliometrics engine using these specific terms (Lortie, 2020d). The focus here was on learning, online, and evidence for an integrated signal of concepts critical to conferencing effectively—in terms of organization, presentations, and engagement. Earlier conceptual work proposes a critical thinking framework for conference‐style learning that happens organically at conferences that can also be applied to classrooms (Underwood & Wald, 1995). Critical thinking, that is directed, purposeful, and active engagement with ideas (Halpern, 2001), is common at conferences with time for questions after oral presentations, poster sessions that now frequently include lively one‐on‐one and group discussions, and panels at many ecology and evolution conferences explicitly designed to tackle learning and dynamism with evidence and ideas in our fields. We need to promote this intellectual discourse when conferences are online and virtual too. Key elements identified in this active learning style at conferences are managed risk, space for participants/attendees to speak, and reminders by moderators that the purpose is not to be negative about the work of others but critical in constructive and positive capacities to better understand the research (Underwood & Wald, 1995). This suggests that online conferences need to include accessible content through recordings and slide decks and that discussion should be moderated with a clear code of conduct (Table 1, items 1–4). This active engagement with speakers is both a form of professional development and networking important to many faculty and researchers (Becerra et al., 2020; Sousa & Clark, 2017), and there is interest in conference‐based online social networking provided wisdom and privacy are promoted—that is, imagine if the conference website was more like a blog and less like a list of static timetables (Levy et al., 2016). Effective engagement through learning at conferences can build new opportunities for research capacity and partnerships with environmental managers and stakeholders (Cooke et al., 2017), and online formats can be even more effective than in‐person because of remote collaboration and reduced to no travel costs (Julsrud et al., 2014). It has been further proposed that critical thinking, creativity, and networking can be enabled through gamification at academic conferences (Castronova, 2013). Gaming and less formal interactions can be developed as mechanisms to promote academic work at conferences because they can be used to provide rewards, increase social networking by having fun together, and if discovery and review are included in the games, promote scientific advances (Castronova, 2013). Shorter talks such as PechaKucha (slide deck auto‐advances and there are a fixed number of slides, that is, 20 slides × 20 s each) and lightning formats (fixed time, short duration talks) are also more fun because of the timed component, both for the presenter practicing and for the listener (Lortie, 2017). Shorter talks will also reduce the cognitive load imposed by attention span issues for content presented via video (Blum, 2020; Bradbury, 2016; Lorenz‐Spreen et al., 2019). With closed captioning or transcription, online recorded talks can promote more individualized learning experiences and accessibility than in‐person rapid talks (Table 1). Recorded talks can be paused and rewinded. Consequently, these contributions can become a form of asynchronous e‐learning that attendees self‐manage remotely with a conference platform tool (Nedeva et al., 2014). Access without the etiquette required for in‐person conferences can be a further benefit (Peté, 2012) thereby reducing potential social barriers.

**Table 1:** A list of proposed innovations for better learning at online conferences in ecology and evolution

Item	Innovation	Description	Learning benefits	Concrete example
1	Provide a code of conduct	This needs to be provided for every meeting in every context. Develop transparent recommendations for commenting and discussion, and we can co‐learn. This is also an opportunity to respect and enable representation and diversity. Volunteers and moderators can promote and monitor online discussion and support positive dialogue	Conference‐style learning can be enabled through critical thinking and discourse online including chat and commentary. However, discussion and comments should be moderated, and a clear code of conduct should be provided in advance of meetings (and ideally agreed upon before commenting is enabled)	The Carpentries Code of Conduct is a community‐developed example for online learning and collaboration (https://docs.carpentries.org/topic_folders/policies/code‐of‐conduct.html)
2	Publish data prior to conference	Online conferences can function as a means to advance open science by providing incentives to share any scientific products individuals are able to provide in advance of meeting (data, data snippets, code, workflows, or syntheses) depending on their respective points in career, degree, or research process	Engagement with data, code, workflows, and other aspects of work that presenters do in advance of their presentations enables deeper learning by the community and advances better understanding of the process of science in addition to the final products such as slide decks, posters, or publications. The sharing can be to the extent that the presenter is comfortable, and it will facilitate better conferences in future because we can see the diversity of ways that others prepare and work	F1000, GitHub, Zenodo, and other online platforms support sharing code and workflows. The resource re3data provided by DataCite provides a comprehensive list of thousands of open data repositories https://www.re3data.org
3	Virtual poster sessions	Virtual poster conferences provide access to the poster digitally in advance of the scheduled session to facilitate review of poster. Then, synchronous Q & A blocks are designated for the presenter to meet attendees	This is a profoundly useful mechanism to engage with the research and ideas of others asynchronously and through re‐engagement with a poster over time. At in‐person sessions, it is sometimes a challenge socially or depending on number of visitors to find a way to ask questions or even see a poster. Accessibility through virtual posters can be promoted and discussion enabled through specific designated times for presenters	The Entomological Society of America and other conferences offer entirely virtual poster conferences (https://www.entsoc.org/events/annual‐meeting)
4	Prerecorded short talks	Reduce all talks to no more than 20 slides, 6 min, prerecorded, and post online. Allow individuals to post whatever content they are comfortable providing (deck only, deck with audio or notes, deck with video, etc)	Rapid presentations can be more compelling, more easily reviewed, reduce cognitive load on attention spans with screens, and often more accessible. Transcription and closed captioning can increase the transparency, clarity, and accessibility of this format. Finally, PechaKucha and Lightning formats are also more fun because they add a simple element of gaming because of timing and pacing challenges	Short talks are supported and hosted by the global collective entitled PechaKucha (https://www.pechakucha.com)
5	define a common slide deck repository	Ideally, identify and provide support for open access slide deck sharing that can be accessed from a browser with desktop and mobile formats supported	Benefits include citable objects, common tags, persistence, and a set of standards for file formats and nomenclature	F1000, Figshare, and others provide a digital online identifier (DOI) for these items similar to publications. Speakerdeck and Slideshare are common platforms (i.e., https://speakerdeck.com)
6	Each session co‐authors a collaborative preprint	Identify two leads per contributed oral session/symposium, and all presenters co‐author a short preprint for session and publish to EcoEvoRxiv	A collaborative paper will promote networks and collaboration. This preprint can also be an important form of synthesis wherein the speakers explore trends and common themes across presentations	The Center for Open Science supports preprints for many domains of science including ecology and evolution (https://www.cos.io/products/osf‐preprints)
7	Provide extemporaneous virtual meetings	It is fantastic to bump into people at meetings. Provide a tool in some format and define mingling times. Every meeting should have this whether in‐person or remote	Discourse can be unstructured and this can engender new ideas through brainstorming and divergent thinking. These processes are critical for discovery in ecology and evolution. Health, child care, accessibility, costs of food, social pressures to meet in bars or different contexts can be addressed with this tool. We can be more inclusive	Numerous tools support meetups including Google Hangouts, Microsoft Teams, Qigochat, Slido, and uMeeting
8	Stream live workshops	Test and plan your specific tool to stream live presentations and collaborative work during the conference. Consider bandwidth and options for streaming at varied resolutions	Sharing not only the final product of our science but how we work is an important learning opportunity. Workshops to show experts solving problems directly teaches and instructs participants at conferences	Use Zoom, Twitch, or any platform to enable and stream live coding, writing, training, and discovery events
9	hackathons or kaggles	Define and plan a kaggle and a few hackathons. We can collaborate and make the presentation process less one‐way and fixed and much more interactive	Many attendees co‐work together at tables at conferences and these opportunities for collaboration can be formalized using online tools with much bigger online tables or workspaces	There are many sites to enable data discovery and collaboration including Kaggle (https://www.kaggle.com/feraco/ecology) and HackEarth (https://www.hackerearth.com/challenges/hackathon/)
10	Capitalize on social media (appropriately)	Use social media more effectively. Predesignate tags, run a photography contest on Instagram from your study work that you present, consider a code snippet sharing event, or run a cartoon or outreach contest	Conference backchannel discussions online at all conferences are an increasingly common means to share notes, ask questions, and highlight common trends	Twitter is a common tool in ecology and evolution but there are other more privacy friendly alternatives including Mastodon, Care2, and Ello

Note

There is a total of 10 items developed using research on meetings and learning and from existing “ten simple rules” publications for online meetings. The innovation is a practice to consider for online conferences, and the description provides a rationale for the proposed concept. Learning benefits describes how the implementation can engender better learning when organizing, presenting, or participating in an online conference. The focus of these innovations was to align some of the open science movement and contemporary practices in ecology and evolution publishing with how we implement conferences.

Finally, the general meta‐science literature associated with online learning at conferences also included synthesis publications to explore acceptance and efficacy of technology. The two key findings are not necessarily qualifiers to the benefits of online conferences for better learning but a reminder that engagement online is not without friction and costs. Acceptance of technology in a systematic review was modeled using intent, perceived ease of use, and perceived benefits (Scherer & Teo, 2019). We now have the intent and context needed to more heavily invest in online conferences, but we must work to ensure that the buy‐in to use the technology is reasonable for both sides of conference participants, that is, organizers/presenters and attendees to actively contribute novel content and commentary. We must further strive to maximize benefits not just in attendance but in learning and consolidation with positive, mediated social connections (Greenhow et al., 2019). The second key finding reported in a relevant meta‐analysis was that webinars work (Gegenfurtner & Ebner, 2019). A contrast of webinars versus online synchronous learning and face‐to‐face instruction showed that webinars were net positive in terms of learner outcomes and marginally more effective in 15 independent contrasts summarizing between 500 and 700 participants (Gegenfurtner & Ebner, 2019). However, there were numerous moderators including duration, instruction format, technology, and level of training of participants. This is not to suggest that webinars should replace all conference presentation formats but that learning at conferences can be enhanced through recording some presentations that are more instructive in nature mimicking lessons used in educational contexts. Collectively, this synthesis evidence suggests that online conferences are viable learning platforms, have many advantages, and provide an opportunity to innovate on how we learn and share findings in ecology and evolution. Notwithstanding, we must carefully plan (and ideally test) deployment and use strategies for our community.

## 3 INNOVATIONS AND APPLICATIONS SPECIFIC TO ECOLOGY AND EVOLUTION

The general science of conferences and webinars provides a clear roadmap to better learning through online conferences. Guidelines from experts and practitioners can provide further insights into many aspects of the academic and scientific endeavor. To this end, there are also a total of eight PLOS Computational Biology “ten simple rules” publications describing best practices directly associated with online conferences (Lortie, 2020b), but there are many more relevant to scientific communication including how to give effective science talks and posters (Bourne, 2007; Erren & Bourne, 2007). The composite list of the 80 simple rules from the immediately relevant editorials supporting online conferences can function as a selective checklist for organizers, presenters, and participants (list published as open dataset: Lortie, 2020b). Here, the simple rules were further classified into one of five following categories: equity, technology, scientific communication, learning, and planning (Appendix S1). Some rules can be classified into multiple categories. For instance, a rule associated with planning for balanced representation in speaker diversity before a conference was classified as planning but also advances equity of presenters and possibly subsequent attendees. Consequently, both the primary intended function of the rules and their secondary classifications were examined. This was an exercise in simplification and was not meant to be prescriptive but descriptive. The goal was to provide a set of earmarks for all these rules but others are possible too (and the compiled list as data is openly available for further exploration). All primary functional groupings for rules were very clear, and the equity category was a broad term intended to capture diversity and inclusivity considerations (Appendix S1). Rules describing planning best practices for conferences were the most frequent classification (code and analyses: Lortie, 2020a). Rules for better learning was the second most common primary function. A secondary classification of the rules (or the indirect benefits of a proposed rule) showed that rules promoting equity dramatically increased in prevalence suggesting that effective planning first will ensure that conferences are well‐prepared to provide both a climate of learning and greater inclusivity. The purpose of this exercise was to cursorily examine how to use the rules for online conferences within the scaffolding of learning and to increase ease of use as a checklist. These editorials encompassed rules for gender balance at conferences (Martin, 2014), how to organize a virtual conference anywhere (Gichora et al., 2010), nonreal‐time conferencing (Arnal et al., 2020), and how to deliver bioinformatics training across the globe (Carvalho‐Silva et al., 2018). Rules included how to make training materials findable and accessible online (Garcia et al., 2020), planning a webinar series, (Fadlelmola et al., 2019), an unconference (Budd et al., 2015), and live tweeting conferences (Ekins & Perlstein, 2014). Nonetheless, there is a still a space to explore how to best apply these concepts in ecology and evolution as organizers, participants, and presenters.

Specifically, in ecology and evolution there is an evolving community of practice of how we work with evidence and collaborate and a culture that to an increasing extent relies on computational tools (Markowetz, 2017) including R (Lai et al., 2019). Thus, there are many distributed and virtual collaboration opportunities. We are working to surface more of the scientific process in ecology and evolution in publishing (Byrnes et al., 2014) through open science, and the same principles can be applied to our conferencing activities. Open science is the movement to make more of the scientific process open and accessible to the public (Allen & Mehler, 2019). This has the benefits of increased transparency, accountability, and higher‐levels of reproducibility in our work (McNutt, 2014). It also produces a wider set of evidence for review, discourse, and learning materials at conferences. A key innovation to consider would be a collaborative contribution by all presenters within a specific session at a conference (Table 1, item 6) such as a preprint to ensure rapid dissemination on a server such as EcoEvoRxiv or bioRxiv. Working together at conferences and actively learning already happens in many other ways at in‐person conferences. It is frequent to see groups of attendees working together at conferences in the hallways and at small tables. We can effectively enable this at online conferences with even larger, open and closed, virtual workspaces that provide extemporaneous, learner‐led forums (Table 1, item 7). Workshops before, and now during, many ecology and evolution conferences are common. We can similarly provide this via live streaming instruction or even more collaboratively using hackathon models (i.e., collective live coding) and online sharing tools such as GitHub to address challenges and co‐work together to collect data, generate ideas, or solve problems (Table 1, items 8–9). Social media at conferences is another important mechanism for collaboration that is common at both in‐person and online conferences (Greenhow et al., 2019; McKendrick et al., 2012). Twitter is a prime example that can provide a medium to advance learning through backchannel discussion to share notes, ask questions, and highlight common trends (Table 1, item 10). This is not a novel online innovation for ecology and evolution, but it can provide an additional postconference opportunity for scientific synthesis and scientific communication if the conference identifies and uses common tags (Ediger et al., 2010). It is one of the ways we can share notes electronically and debrief. It can be even more useful when the conference is entirely online and we are on devices already. This further suggests that many of the online products and artifacts of a conference in addition to the traditional conference abstracts can be used in future research if online conferences in ecology and evolution develop archival plans for materials (similar to a data management plan) and ensure open and accessible persistence. An online conference can become a learning hub after the formal meeting is over.

## 4 IMPLICATIONS

We are faced with a challenge and an opportunity. Online conferences can enable better learning in numerous respects relative to traditional in‐person meetings with sufficient planning and foresight. This is will be a process of experimentation, and the research and rules summarized here can function as a checklist for planners. The evidence strongly suggests that collecting data at online conferences and formalizing feedback from participants will be a major path forward for much better conferences in future (Arnal et al., 2020)—in spite of what we test this year and next out of necessity. The research and publishing world is evolving in ecology and evolution (perhaps not quickly enough), and we now have the impetus to rapidly align another relatively major component of the scientific pursuit, conferences, or to pioneer new directions. Networks are a part of the community in ecology and evolution, and perhaps online conferences can level some of the existing power dynamics and gender differences (Biggs et al., 2018; Peté, 2012) and build new networks. We can also broaden our reach outside the immediate academic community with some components of online conferences made fully accessible to the public. Conferences are a commitment, and we can commit to making them better (and even more engaging).

## CONFLICT OF INTEREST

CJL declares no conflict of interest.

## AUTHOR CONTRIBUTION

**Christopher J Lortie:** Conceptualization (lead); Data curation (lead); Formal analysis (lead); Investigation (equal); Methodology (lead).

### OPEN RESEARCH BADGES

This article has been awarded Open Data and Open Materials Badges. All materials and data are publicly accessible via the Open Science Framework at Figshare: https://figshare.com/articles/A_compiled_meta‐list_of_ten_simple_rules_for_better_online_conferences/12493187/3 and Codebook on Zenodo: https://zenodo.org/account/settings/github/repository/cjlortie/BLOC_IT

## Supporting information

Supinfo

## Data Availability

All data and R code are publicly available at open access repositories. Lortie, C.J. (2020) BLOC_IT: Code and data supporting ten simple rules for better learning at online conferences. Zenodo, 1.2, 1–3. Lortie, C.J. (2020) A compiled meta‐list of ten simple rules for better online conferences. Figshare, 1, 1–2. Lortie, C.J. (2020) A list of eeb conferences in 2020. Figshare, 1, 1–3.
