# Supplementary material for: Online conferences for better learning
Source: Ecol Evol. 2020 Oct 22;10(22):12442–9. doi: 10.1002/ece3.6923 (PMC7679531; doi:10.1002/ece3.6923)

**Appendix S1. Online conferences for better learning**

**A compiled list of ten simple rules for better online conferences.**

This is composite meta-list of the rules from 8 PLOS Computational Biology ‘ten simple rules’ publications describing best practices directly associated with online conferences.

The entire dataframe is published online and fully open for reuse including additional classification.

Data here: <https://figshare.com/articles/dataset/A_compiled_meta-list_of_ten_simple_rules_for_better_online_conferences/12493187>

Citation: Lortie, Christopher (2020): A compiled meta-list of ten simple rules for better online conferences. figshare. Dataset. https://doi.org/10.6084/m9.figshare.12493187.v4

**Meta-data**

Rep is the replicate from 1-8 to itemize each editorial for reference.

Paper is the title of the editorials.

Concept is a simple term to describe the key purpose of each editorial.

Rules are always from 1 to 10.

Description is the rule as listed in simple format from each editorial.

Category is a classification proposed to sort the rules into planning, learning, technology, scientific communication, and accessibility.

**Studies**

Martin, J. L. 2014. Ten Simple Rules to Achieve Conference Speaker Gender Balance. PLOS Computational Biology 10.

Gichora, N. N., S. A. Fatumo, M. V. Ngara, N. Chelbat, K. Ramdayal, K. B. Opap, G. H. Siwo, M. O. Adebiyi, A. El Gonnouni, D. Zofou, A. A. M. Maurady, E. F. Adebiyi, E. P. de Villiers, D. K. Masiga, J. W. Bizzaro, P. Suravajhala, S. C. Ommeh, and W. Hide. 2010. Ten Simple Rules for Organizing a Virtual Conference—Anywhere. PLOS Computational Biology 6:e1000650.

Arnal, A., I. Epifanio, P. Gregori, and V. Martínez. 2020. Ten Simple Rules for organizing a non–real-time web conference. PLOS Computational Biology 16:e1007667.

Carvalho-Silva, D., L. Garcia, S. L. Morgan, C. Brooksbank, and I. Dunham. 2018. Ten simple rules for delivering live distance training in bioinformatics across the globe using webinars. PLOS Computational Biology 14:e1006419.

Garcia, L., B. Batut, M. L. Burke, M. Kuzak, F. Psomopoulos, R. Arcila, T. K. Attwood, N. Beard, D. Carvalho-Silva, A. C. Dimopoulos, V. D. del Angel, M. Dumontier, K. T. Gurwitz, R. Krause, P. McQuilton, L. Le Pera, S. L. Morgan, P. Rauste, A. Via, P. Kahlem, G. Rustici, C. W. G. van Gelder, and P. M. Palagi. 2020. Ten simple rules for making training materials FAIR. PLOS Computational Biology 16:e1007854.

Fadlelmola, F. M., S. Panji, A. E. Ahmed, A. Ghouila, W. A. Akurugu, J.-B. Domelevo Entfellner, O. Souiai, N. Mulder, and H. A. R. w. g. a. m. o. t. H. A. Consortium. 2019. Ten simple rules for organizing a webinar series. PLOS Computational Biology 15:e1006671.

Budd, A., H. Dinkel, M. Corpas, J. C. Fuller, L. Rubinat, D. P. Devos, P. H. Khoueiry, K. U. Foestner, F. Georgatos, F. Rowland, M. Sharan, J. X. Binder, T. Grace, K. Traphagen, A. Gristwood, and N. T. Wood. 2015. Ten Simple Rules for Organizing an Unconference. PLOS Computational Biology 11.

Ekins, S., and E. O. Perlstein. 2014. Ten Simple Rules of Live Tweeting at Scientific Conferences. PLOS Computational Biology 10.

**Figure S1. The relative frequency of simple rules supporting better online conferences classified by category.** Rules were classified into si
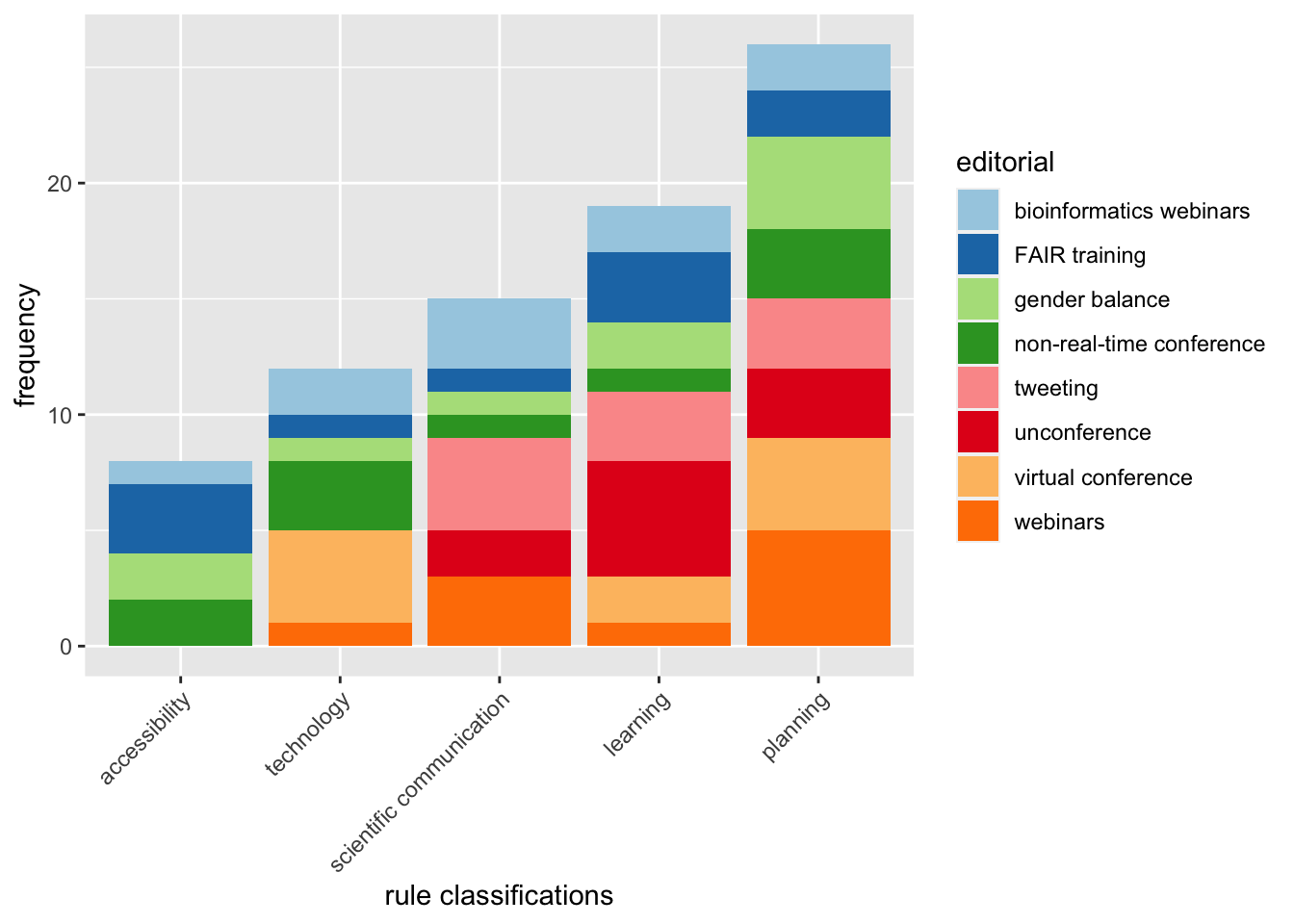
ngle categories only, and code to visualize these data are freely available at Zenodo and GitHub. See text for description and citations.

**Figure S2. The relative frequency of simple rules supporting better online conferences classified by category.** Rules were classified into secondary categories because more than one classification was possible some proposed rules, and code to visualize these data are freely available at Zenodo and GitHub. See text for description and citations. This process of additional classification highlighted that many of the primary functions proposed by the rules can better enable and promote equity and accessibility for participants.


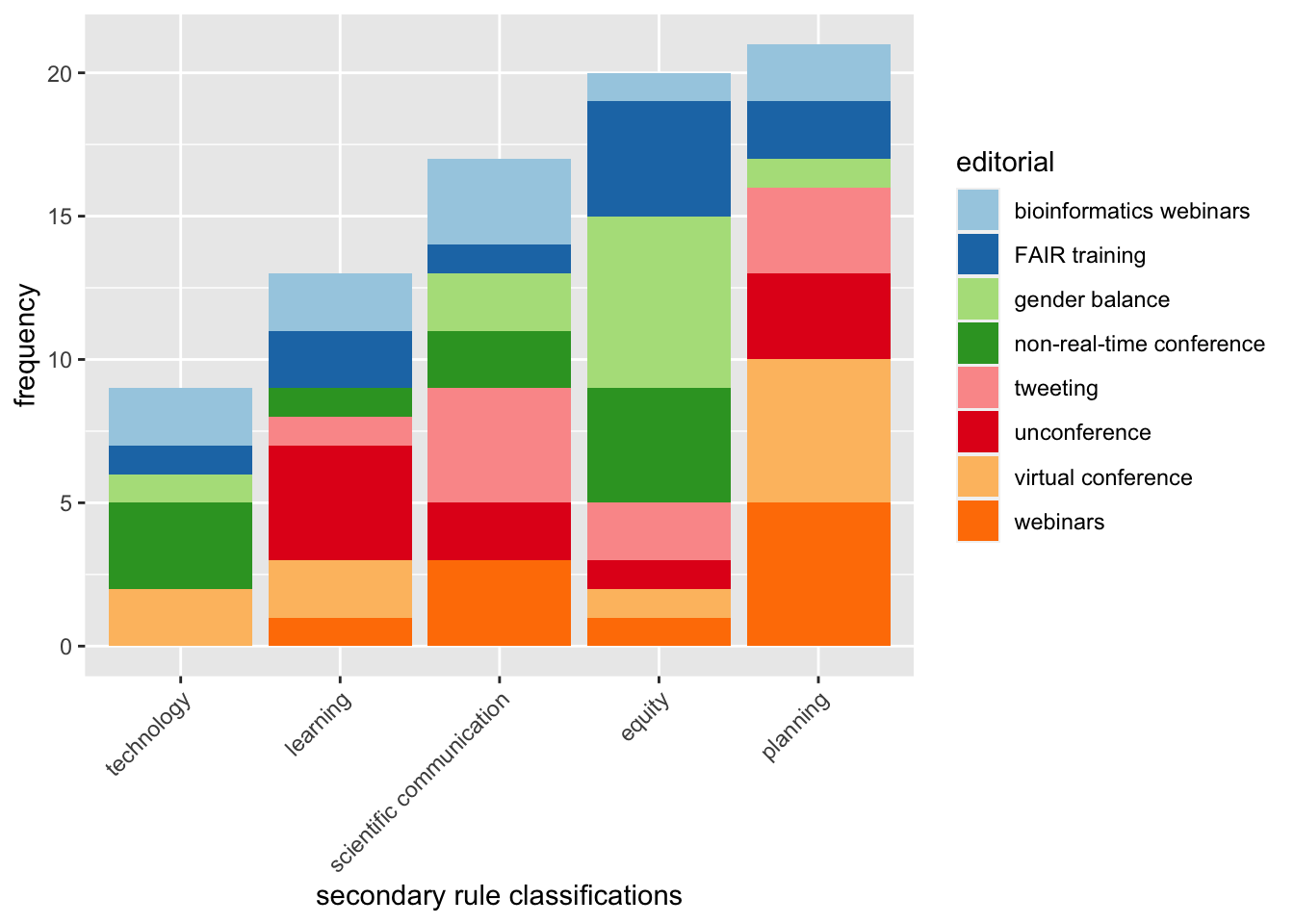

Supplement: Supplementary file 1 — Supinfo [file ECE3-10-12442-s001.docx]
